# Supplementary material for: The Effects of Chronic Consumption of Lipid-Rich and Delipidated Bovine Dairy Milk on Brown Adipose Tissue Volume in Wild-Type Mice
Source: Nutrients. 2021 Nov 26;13(12):4266. doi: 10.3390/nu13124266 (PMC8704458; doi:10.3390/nu13124266)
Supplement: Supplementary file 1 [file nutrients-13-04266-s001.zip › nutrients-1467188-supplementary.pdf]

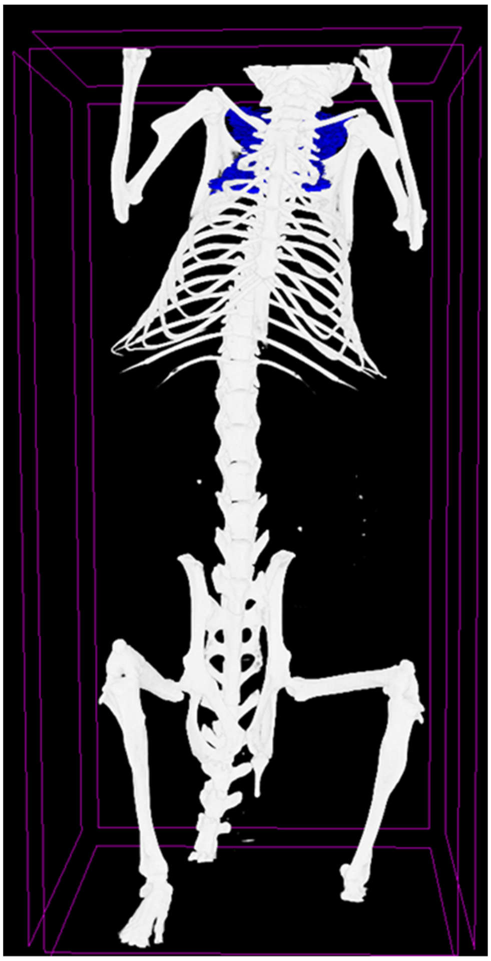

Supplementary Figure S1. Representative SkyScan image of mouse subscapular BAT (blue) from HF+FC group.

Supplementary Table S1. Macronutrient composition of dietary intervention components

	**Diet macronutrient composition (%)**		
	**Lipid**	**Carbohydrate**	Protein
SF07-050 (HF)	20.33	66.12	13.55
AIN93M (LF)	4.0	82.2	13.8
Full-cream milk	3.6	4.9	3.3
Skim milk	0.01	5.0	3.4
